# Human ABO Blood Groups and Their Associations with Different Diseases

**DOI:** 10.1155/2021/6629060

**Published:** 2021-01-23

**Authors:** Silamlak Birhanu Abegaz

**Affiliations:** Woldia University, Faculty of Natural and Computational Sciences, Department of Biology, Ethiopia

## Abstract

**Introduction:**

Human ABO blood type antigens exhibit alternative phenotypes and genetically derived glycoconjugate structures that are located on the red cell surface which play an active role in the cells' physiology and pathology. Associations between the blood type and disease have been studied since the early 1900s when researchers determined that antibodies and antigens are inherited. However, due to lack of antigens of some blood groups, there have been some contentious issues with the association between the ABO blood group and vulnerability to certain infectious and noninfectious diseases.

**Objective:**

To review different literatures that show the association between ABO blood groups and different diseases.

**Method:**

Original, adequate, and recent articles on the same field were researched, and the researcher conducted a comprehensive review on this topic. Thus, taking out critical discussions, not only a descriptive summary of the topic but also contradictory ideas were fully retrieved and presented in a clear impression. In addition, some relevant scientific papers published in previous years were included. The article search was performed by matching the terms blood types/groups with a group of terms related to different diseases. The articles were screened and selected based on the title and abstract presented.

**Results:**

The susceptibility to various diseases, such as cancer, cardiovascular diseases, infections and hematologic disorders, cognitive disorders, circulatory diseases, metabolic diseases, and malaria, has been linked with ABO blood groups. Moreover, blood group AB individuals were found to be susceptible to an increased risk of cognitive impairment which was independent of geographic region, age, race, and gender. Disorders such as hypertension, obesity, dyslipidemia, cardiovascular disease (CVD), and diabetes were also more prevalent in individuals with cognitive impairment. Early etiological studies indicated that blood type O has a connection with increased incidence of cholera, plague, tuberculosis infections, and mumps, whereas blood type A is linked with increased incidence of smallpox and *Pseudomonas aeruginosa* infection; blood type B is also associated with increased incidence of gonorrhea, tuberculosis, and *Streptococcus pneumoniae*, *E. coli*, and salmonella infections; and blood type AB is associated with increased incidence of smallpox and *E. coli* and salmonella infections. Diabetes mellitus, hypercholesterolemia, arterial hypertension, and family history for ischemic heart disease are the most common risk factors for cardiovascular diseases and can be genetically transmitted to offspring. Higher incidence of cancers in the stomach, ovaries, salivary glands, cervix, uterus, and colon/rectum was common in blood type A people than in O type people. The link between the ABO blood type and thromboembolic diseases and bleeding risk are intervened by the glycosyltransferase activity and plasma levels and biologic activity of vWF (Von Willebrand factor), a carrier protein for coagulation factor VIII which is low in O type.

**Conclusion:**

Several studies related to the ABO phenotype show that genetically determined human ABO blood groups were correspondingly linked with an increased risk of various infectious and noninfectious diseases. However, further investigations are needed particularly on the molecular level of ABO blood groups and their association with various diseases.

## 1. Introduction

Human ABO blood type antigens exhibit alternative phenotypes and genetically derived glycoconjugate structures that are located on the red cell surface which play an active role in the cells' physiology and pathology [1, 2]. Furthermore, oligosaccharide structures specific to the antigens defined the blood type. Thus, blood group antigens are secondary gene products, whereas various glycosyltransferase enzymes that help attach the sugar molecules to the oligosaccharide chain are primary gene products. These carbohydrate components are perceived as extraneous by the immune system of others and produce antibodies to them [3]. Medically, the ABO blood group system has been of great importance in different disease studies [4]. However, due to lack of antigens of some blood groups, there have been some contentious issues with the association between the ABO blood group and vulnerability to certain infectious and noninfectious diseases. The presence and absence of antigens in some blood types result in blood membrane alterations in both morphology and function. The functions dependent on the structure of blood types can associate the blood groups with diseases as well as health [1, 5]. In addition to RBCs, blood group antigens can be found on leukocytes, certain tissues, plasma proteins, platelets, and various cell surface enzymes [6]. Blood group antigens can also be present in body fluids such as sweat, saliva, breast milk, seminal fluid, urine, gastric secretions, and amniotic fluid with soluble form [7, 8]. Most antigens however are the end product of a single gene, and modifications at the genetic level such as deletions, inversions, insertions, alternative splicing, or single-nucleotide polymorphisms (SNPs) lead to antigenic changes but can also give rise to new antigens or even complete loss of expression [3]. The ABO blood group antigens are possibly the most significant and intensively studied and were the first to be recognized [9]. The glycoconjugate structures on RBCs have many functions including receptors for exogenous ligands, viruses, bacteria, and parasites, transporters, channels, structural proteins, adhesion molecules, and enzymes [10]. The exact mechanisms that would explain the associations between blood group antigens and disease in adhesion molecules however have not yet known. But an unexpected number of these structures contribute to normal RBC development; some act as cell adhesion molecules (CAMs), and some play a role in human disease [11, 12]. The ABO, Hh, Sese (secretor), and Lele (Lewis) genes play different roles in the final ABO antigen structure of an individual's body tissues as well as secretions [6, 7]. However, this basic evidence can be revised as new information was obtained [13]. The ABO blood group has been associated with many diseases and easily accessed in a patient's genetic makeup [14]. In the years 1960 and 1970, large epidemiological studies were carried out around the world and connections between the human ABO blood group and vulnerability to develop a number of diseases were broadly postulated [15]. ABO blood types were shown to have some associations with various infectious [7, 16] and noninfectious diseases [7, 17]. Environmental and host genetic factors may be imperative in the genesis of diseases. During 1901 Landsteiner's discovery, the ABO blood group system was studied as an etiological factor of many diseases, for example, carcinoma of the stomach and peptic ulcer [18]. ABO antigens are thought to be evolutionarily beneficial in conferring resistance against pathogens. However, the susceptibility to various diseases, such as cancer, cardiovascular diseases, infections, and hematologic disorders, has been linked with ABO blood groups [19]. Mostly A and B antigens are secreted by the cells and are found in human blood circulation. Therefore, nonsecretors are at risk to a variety of infections [20]. The likely pathogenesis for this vulnerability is that many organisms may bind to polysaccharides on the cell surface but soluble blood group antigens may block this binding [16, 21]. On the one hand, [22] reported that the ABO alternative phenotype can prevent the species carrying it from being threatened by a pathogen using a given carbohydrate as a receptor. On the other hand, the ABO polymorphism leads to a polymorphic production of anti-A and anti-B natural antibodies, which potentially protect individuals from several infectious agents expressing A and B motifs [23]. In view of the above possible associations of the ABO blood group and different diseases, the objective of this paper is thus to review different literatures that show the association between ABO blood groups and different diseases.

## 2. Methodology

Original, adequate, and recent articles on the same field were researched, and the researcher conducted a comprehensive review on this topic. Thus, taking out critical discussions, not only a descriptive summary of the topic but also contradictory ideas were fully retrieved and presented in a clear impression. In addition, some relevant scientific papers published in previous years were included. The article search was performed by matching the terms blood types/groups with a group of terms related to different diseases. The articles were screened and selected based on the title and abstract presented.

## 3. Association of ABO Blood Groups and Diseases

### 3.1. Infectious Diseases

According to the study of [24–26], ABO polymorphism has been associated with certain infectious diseases. The presence/absence of A/B antigens and correspondent absence/presence of anti-A/B antibodies provide strong/weak defensive lines against infection. Many vertebrate species uphold the ABO gene and hence benefited from it. However, having both functional A and B genes in common within species may not be important because they may lose anti-A/B antibodies in the long run. But frequent gene conversion of A/B specificity producing amino acid substitutions or recombination with nonfunctional partial genes may have conferred an adaptation against microbial attacks [27]. Glycoconjugate red cell surfaces are used effectively by parasites, bacteria, and viruses as receptors for attachment [9]. Because infectious agents often use cell surface glycoconjugates as receptors for attachment, glycosylation polymorphisms in the ABO blood type may affect host-pathogen connections and result in vulnerability difference among individuals with diverse glycosylation profiles [9]. It should be recalled that certain microbial parasites share blood group antigens with their hosts (molecular mimicry). An association between ABO and infectious diseases like cholera was identified by early etiological studies [9]. According to the study of [28], the major variations observed in ABO blood groups in different parts of the world were due to the epidemics that have occurred in the past. The presence of an “H-like” antigen on the plague bacillus (and cholera) and an “A-like” antigen on the smallpox virus was suggested as some major differences. This would make individuals who can make anti-H (A1 and B) more resistant to the plague and cholera and individuals who have anti-A (groups B and O) more resistant to smallpox [1, 29]. Reports showed that once a person is infected with cholera (*Vibrio cholerae* strains O1 El Tor and O139), the O blood types had a greater frequency of severe infections than the non-O blood types [26]. Patients of blood type O were more vulnerable to infections like gastrointestinal outbreaks caused by *Escherichia coli* O157 in Scotland in 1996, and a total of 87.5% of blood type O patients died. According to the study of [30], blood type O was also associated with increased incidence of cholera, plague, tuberculosis infections, and mumps, whereas blood type A was associated with increased incidence of smallpox and *Pseudomonas aeruginosa* infection; blood type B is also associated with increased incidence of gonorrhea, tuberculosis, and *Streptococcus pneumoniae*, *E. coli*, and salmonella infections; and blood type AB is associated with increased incidence of smallpox and *E. coli* and salmonella infections. The Norovirus, a strain-dependent pathogen, however, is likely to have less risk of infection and symptomatic disease in blood group B individuals, but blood group O individuals have a much greater risk of infection [1, 9]. The expression of Le and ABH antigens in the gastrointestinal tract is also closely linked to vulnerability of Norovirus infection. Nonsecretors appear more susceptible to infections by *Haemophilus influenzae*, *Neisseria meningitides*, [7, 30], and *Streptococcus pneumoniae* and urinary tract infection caused by *E. coli* [31]. Peptic ulcer also has a connection with the ABO blood group, and it was the first to be identified. Blood type O individuals showed that they had higher susceptibility to peptic ulcers [1, 9]. Gastritis and ulceration of the stomach/duodenum were later correlated with infection with the bacterium *Helicobacter pylori*. It was also found that antibiotics and acid secretion inhibitors cured patients from peptic ulcer by eradicating the bacteria causing it [30, 32]. It was reported that *H. pylori* attachment to the human gastric mucosa was mediated by the H type 1 and Lewis b (Le^b^) fucosylated antigens. The soluble glycoproteins presenting Le^b^ or antibodies to the Le^b^ antigen could inhibit the bacterial binding. Furthermore, the conversion of Le^b^ to ALe^b^ by the addition of a terminal GalNAc weakened the bacterial binding. This may explain the decreased infectivity of blood type AB/B/A individuals as compared to blood type O individuals [7, 9]. Infections related to bacterial meningitis is another example of infectious disease which can be linked to the ABO phenotype. Three species of bacteria, *N. meningitides*, *H. influenza*, and *S. pneumoniae*, cause about 75% of all bacterial meningitis; the capsules of these bacteria contain polysaccharide antigens, which the immune defense of the host must recognize and respond to with the appropriate antibodies [7, 33].

### 3.2. Cognitive Disorders

Higher levels of plasma coagulant glycoproteins, vWF (Von Willebrand factor), and FVIII (factor VIII) in non-O blood group individuals have been connected with greater risk of dementia and cognitive impairment, suggesting that coagulation factors may play a role in these disorders [7, 34]. Blood group AB individuals were found to be susceptible to an increased risk of cognitive impairment (OR = 1.82) as revealed by a large prospective case-control study, which was independent of geographic region, age, race, and gender [7]. In this study, the FVIII levels differed significantly among blood groups with the order AB > B > A > O [7, 35], and disorders such as hypertension, obesity, dyslipidemia, cardiovascular disease (CVD), and diabetes were also more prevalent in individuals with cognitive impairment, which points out that a common etiology is likely; the blood group influences CVD risk, and CVD risk factors are known to be connected with dementia and cognitive impairment. Surprisingly, blood types AB and B were more frequent in Blacks (in both cases and controls) [7, 35], which may be an overlooked factor in increased rates of stroke and CVD in this population.

### 3.3. Circulatory Diseases

Factually, several studies reported that non-O blood groups are linked with the high risk of ischemic heart disease and emergence of atherosclerosis indicators [36]. Moreover, peripheral vascular disease, venous thromboembolism, myocardial and angina infarction, and these connections were checked by subsequent GWAS (genome-wide association studies) and further confirmed in 2008 with a systematic review and meta-analysis [1, 37]. Genetic factors also contribute to the development of coronary artery disease (CAD) and influence individual response to risk factor modification. The most significant cardiovascular risk factors, diabetes mellitus, hypercholesterolemia, arterial hypertension, and family history for ischemic heart disease, are, at least in part, genetically transmitted. Of these, family history very accurately predicts coronary events [1, 38]. ABO blood types are genetically transferred as well, through chromosomes 9 at locus 9q34. The ATP-binding cassette 2 (ABCA2) genes were located at locus 9q34 which plays a role in cholesterol homeostasis. Furthermore, genetic variation considerably affects the risk for developing CAD which has been discovered recently on the 9p21 chromosomal section. Much of the hereditary aggregation of CAD can be linked to genetic risk factors. Therefore, inheritance of ABO blood types could play a significant role in this context [9, 38]. ABO blood type antigens are connected to the protein backbone of coagulation factors vWF and FVIII and hence undoubtedly affect coagulation. To be sure, blood type O patients are prone to excessive blood loss because of just about 25% lower concentrations of vWF and FVIII coagulation factors in the plasma, which is the result of the increased avoidance of these glycoproteins; the phenomenon is related to the H antigen linked to their backbone [26]. On the other hand, the higher plasma concentrations of coagulation factors vWF and FVIII in non-O blood type persons have been connected with the greater risk for ischemic heart disease and thromboembolic disease [1, 26]. Blood type AB patients with preeclampsia have an increased risk of severe, early-onset, or intrauterine growth restriction (IUGR) forms. Preeclampsia, the leading cause of maternal and fetal morbidity and mortality during pregnancy, was unique and likely to be associated with maternal blood type. Placental protein 13 (PP13) is a galectin (galectin-13) that binds to beta-galactosides like N-acetyl-galactosamine, galactose, and fucose, located at end positions on ABO blood type antigens. PP13 is also reflected as an early marker for preeclampsia. PP13 is mainly produced by the placenta in anthropoid primates and is predominantly localized to the syncytiotrophoblast apical membrane, where it can be secreted and/or shed into the maternal circulation [19]. Similarly, ABO was linked to myocardial infarction [9].

### 3.4. Cancer

Even though the association between the ABO blood type and cancer was the subject of rigorous investigation in the mid-1900s, there has been improved attention after the contemporary publication of reports establishing a connotation between the ABO blood type and pancreatic cancer [39–41]. Blood type antigens play a role in tumorigenesis, metastasis, and prognosis and are probably take part in cell recognition, cell signaling, and cell adhesion [42]. Expression of ABH and related antigens differs during cellular development, aging, and differentiation; this is mainly realistic during carcinogenesis and pathological phenomena [9]. ABH antigens can be found in the epithelial tissues of the gastrointestinal tract, breast, uterine cervix, mouth, lung, bladder, and prostate. But these antigens are missing from the glycolipids and glycoproteins of malignant tissues in these areas [31]. For example, it is thought that DNA methylation in the promoter region for the blood group A gene may inhibit transcription of the associated enzyme and therefore loss of the A antigen, but different mechanisms for the reduction of mRNA have been found in A tumors, which appear to be specific to each tumor cell line [43]. Loss of A and B antigens leads to metastasis, resulting from downregulated transcription of ABO with related loss of A or B transferase activity, and upsurges the buildup of other antigens which act as ligands for selectins and facilitate the metastatic process [31]. As normal antigens are lost, malignancy develops and tumor antigens are acquired; the decrease in A, B, and H antigens is indirectly proportional to the metastatic potential of the tumor [30]. Procoagulant and angiogenic properties of blood type antigens are known, act as ligands for selectins, and increase cellular motility and resistance to apoptosis; these biological roles may assist tumor development, and a model has been proposed that may account for the described relations between the presence or absence of these markers and the outcome of disease [44]. Some non-A blood type people have tumors with real A antigens or with “A-like” antigens that have very similar properties to A antigens; in these people, the tumor antigens would be seen as foreign and would interact with anti-A antibodies, resulting in attack of the tumor [7, 30]. This explains why a greater incidence of cancer is common in blood type A people than in blood type O people; the A or “A-like” properties of these tumor antigens are not recognized as foreign in blood type A people [30]. Blood type A people have a greater occurrence of cancer in the stomach (22%), ovaries (28%), salivary glands (64%), cervix (13%), uterus (15%), and colon/rectum (11%) as compared to blood type O people [7, 30]. It is important to note that although ABO genotypes are considerably associated with the risk of certain cancers, they do not cause cancer; they only indicate susceptibility [9]. In the reverse, lack of association does not confer protection: many studies have been unsuccessful to find the link between the blood type and breast cancer [7, 45, 46].

#### 3.4.1. Leukemia and Lymphoma

A and B antigens were usually decreased till they are untraceable in patients with acute leukemia and aplastic anemia; as the patient's condition improves, the antigens increase again to their former levels [30]. However, the loss of antigens may be due to an inhibitory factor related to antigen-antibody binding or an abnormal distribution or density of antigen sites in the RBC membrane but not deficiency in transferase synthesis or activity [7, 47]. Expression of A, B, or H antigens in leukemia patients had significantly lowered between 17% and 37% when compared to healthy controls of A, B, or AB. Patients with myeloid malignancies had reduced expression of A or B antigens. Blood type O patients had reduced H antigens by 55% and 21% when compared with healthy controls of the same ABO genotype [31]. Primary central nervous system lymphoma (PCNSL) begins in and characteristically remains limited to the central nervous system (CNS) in non-Hodgkin's lymphoma [7, 48], while secondary central nervous system lymphoma (SCNSL) characteristically does not originate from the CNS but may later take part in the CNS up to 10% to 30% of cases [7, 49]. A study of 36 patients with PCNSL occurrence reported 8.3% in blood type A, 27.8% in blood type B, 55.6% in blood type O, and 8.3% in blood type AB [48] whereas another study that assessed 202 patients with secondary central nervous system lymphoma (SCNSL) indicated that the occurrence was 5.0% in blood type A, 61.9% in blood type B, 29.7% in blood type O, and 3.5% in blood type AB [7, 49]. The same populations of healthy controls were also used for both studies; the shared blood type percentages were 22.2% in blood type B, 37.1% in blood type A, 6.1% in blood type AB, and 35.6% in blood type O [48, 49]. There are very few studies of the association between ABO blood types and children with leukemia and lymphoma. A study of pediatric victims with acute myeloid leukemia (AML; *n* = 116), acute lymphoblastic leukemia (ALL; *n* = 522), Hodgkin's lymphoma (*n* = 63), and non-Hodgkin's lymphoma (*n* = 78) exhibited an important variation in the overall dissemination of blood groups when compared to the source population for all but the AML victims [7, 48]. This study stated that the occurrence of Hodgkin's lymphoma was 45.6% greater in blood type B victims and 56.5% lesser in blood type A patients; the occurrence of non-Hodgkin's lymphoma was 52.9% lesser in blood type A patients; the occurrence of ALL was 14.3% greater in blood type O victims; but there was no variation in the dissemination of blood types in victims with AML [7, 49]. A distinct multicenter pediatric study of 682 victims with ALL and 224 victims with AML stated that the occurrence of ALL was 56.5% greater in blood type O victims, 35.8% lesser in blood type A victims, and 26.9% lower in blood type B victims, while the occurrence of AML was 28.8% greater in blood type A victims [7].

#### 3.4.2. Stomach Cancer

It is the fourth most public cancer worldwide and the second leading cause of cancer deaths; reliable studies since the 1950s have shown that blood type A persons have about a 20% higher risk of stomach cancer than blood type O individuals [50]. The 2012 metadata analysis showed that type A persons had an odds ratio of 1.11 and type O persons had an odds ratio of 0.91 for gastric cancer; in addition, blood type A persons had significantly greater rates of *H. pylori* infection than non-A blood type victims (odds ratio = 1.42) [7, 51]. This is important because a current study of ABO blood types and *H. pylori* found that the risk of advanced precancerous gastric lesions was considerably affected by the presence or absence in the bacterial DNA of two SNPs in the cytotoxin-associated gene *A* (*CagA*), recognized as *CagA*-positive and *CagA*-negative strains [7, 52]. Thus, ABO antigens on the gastric epithelium are binding sites for the *H. pylori* bacterium, which then injects *CagA* virulence protein into the cellular cytoplasm [7, 52]. In persons diseased with *CagA-*positive *H. pylori*, the danger was considerably greater in blood type A than in blood group O for intestinal metaplasia (OR = 1.36) and dysplasia (OR = 1.78), with a collective OR of 1.42, whereas in those with *CagA*-negative strains or who were not diseased with *H. pylori*, blood type A persons had a considerably lesser danger than blood type O persons for intestinal metaplasia and dysplasia (OR = 0.60) [7, 52].

#### 3.4.3. Pancreatic Cancer

It is the seventh most common cause of cancer death worldwide and is one of the most destructive cancers, with death rates nearly equal to occurrence rates [50]. Blood type A, B, and AB persons have a 25% higher risk of gastric and pancreatic cancer, as well as 17% higher risk of pancreatic cancer only; as compared to blood type O, the vulnerability of exocrine pancreatic cancer is uppermost in blood type B (odds ratio, 1.72) and lower for blood types AB (odds ratio, 1.51) and A (odds ratio, 1.32) [7, 9]. Secretor status had insignificant consequence on this risk, but the behavior of *H. pylori*, which is also influenced by the blood type, may have an effect on risk [7, 31]. Non-O blood type persons diseased with *CagA-*negative *H. pylori* have greater risk for pancreatic cancer (OR = 2.78) [53]. Non-O blood type persons diseased with *CagA*-negative *H. pylori* have greater risk for pancreatic cancer (odds ratio = 2.78) [50]. The greater rate of pancreatic cancer in non-O blood type persons also has been explained by a genome-wide association study [53] which found that the SNP rs505922 mapped to the first intron of the ABO blood type gene in the 9q34 locus and was in complete linkage disequilibrium with the O/non-O allele [7, 45]. Another study also found that blood group O is intensely linked with pancreatic disease in patients with von Hippel-Lindau (VHL) syndrome and is considerably associated with solid pancreatic lesions in victims with pancreatic neuroendocrine tumors (PNETs); VHL victims have a great risk of evolving multiple tumors throughout the body, and their risk of developing both benign and malignant PNETs is 8% to 17% [7, 42].

#### 3.4.4. Multiple Endocrine Neoplasia Type I (MENS-1)

It was detected in 105 patients, among which 46 (43.8%) were identified with a neuroendocrine tumor; from these 46 sufferers, 14 had several forms of tumor for the entire 60 tumors; it was found in the lung (*n* = 5), stomach (*n* = 3), gallbladder (*n* = 1), duodenum (*n* = 13), pancreas (*n* = 34), or thymus (*n* = 4) [7, 42]. Of the 17 patients with metastatic tumors, 16 of them (93.8%) had blood type O, whereas of the 43 benign tumor patients, 32 of them (74.4%) were blood type O; of the 46 neuroendocrine tumor patients, 35 of them (76.1%) had blood type O, 9 of them (19.6%) were blood type A, 2 of them (4.3%) were blood type B, and none were blood type AB. Of the 59 nonneuroendocrine tumor patients, only 31 of them (52.5%) were blood type O, 15 of them (25.4%) were blood type A, 7 of them (11.9%) were blood type B, and 6 of them (10.2%) were blood type AB [7, 42]. Blood type AB is found in 4% of the overall population and was found in 3.8% of the study cohort, so the absence of type AB in patients with neuroendocrine tumors was notable [7, 42].

#### 3.4.5. Cancer of the Rectum/Colon

Augmented activity of the *α*1,4-fucosyltransferase of the *FUT3* gene (Lewis) and *α*1,2-fucosyltransferase of the *FUT2* gene (secretor) seems to take part in the development and regulation of cancers of the distal colon; type 2 as well as type 1 chains and Lewis antigens are generally existing in the fetal colon and vanished in healthy adults, but they reemerge in grown persons who have distal colon cancers [7, 31, 54]. Only type 1 chains are expressed by secretors in the normal colon; however, type 1 or type 2 chains are not common by nonsecretors in normal or cancerous colon tissue [55]. Secretor status also determines whether the H antigen (blood group O) can be seen in normal and cancerous colon tissue [7, 55]. The goblet cell which is the secretory part of the normal colon is crucial for the expression of blood type antigens [54]. Blood type A antigen is rarely seen on malignant tumors of type O or type B persons; about 10% of colon tumors of homozygous type O people express A antigen and have *N*-acetylgalactosaminyltransferase activity [7, 31].

### 3.5. Metabolic Diseases

Since metabolic diseases are multifactorial and are not controlled by a single gene or antigen, the role of blood types in metabolic disease is most likely to be complex. However, some fascinating relationships have been found and presented below.

#### 3.5.1. Hypertension

It can have several causes; thus, it is not surprising that different studies have found various links between blood type antigens and hypertension. Some investigations indicate that the rate of hypertension in blood type B was maximum, followed by blood type A and blood type AB which had the lowest rate of hypertension [56]. Another study also reported that there was a link between blood type A and systolic blood pressure in Caucasians but not in Blacks [7, 57]. In hypertension due to abnormal red blood cells' sodium and potassium transport, no connection was found with the Rh, ABO, Kidd, Duffy, MNS, or P blood types or with the major histocompatibility HLA antigens [7, 58]. In hypertension due to abnormal red cells' lithium-sodium counter transport, no connection was found with the MNS blood type polymorphism [7, 59]. When secondary forms of hypertension can be ruled out, essential hypertension is possibly diagnosed. However, it can also be diagnosed due to atherosclerotic or fibromuscular etiology, renal stenosis (renovascular hypertension), or primary aldosteronism linked with little plasma renin levels; people with these circumstances were compared to normotensive controls and persons with secondary hypertension, and insignificant changes were obtained in ABO, Rh, Kidd, Kell, Duffy, P, haptoglobin, PGM-1, or acid phosphatase systems [56, 58]. Conversely, there were important alterations in the incidences of the MNS blood type antigens when comparing normotensive controls with persons who had essential or renovascular hypertension; when compared to normotensives, essential hypertensives were considerably dissimilar among Whites, whereas the same alteration was not seen among Blacks [58]. Three clearly different phenotypic occurrences were seen when persons with atherosclerotic renovascular hypertension were compared to essential hypertensives and normotensives [56, 58].

#### 3.5.2. Hyperlipidemia

Investigators have also studied the relationship between ABO blood type antigens and hyperlipidemia. Some study stated that LDL cholesterol, total cholesterol, and triglycerides were higher while HDL cholesterol was lower, in blood types A and B; however, blood type AB was protective for hyperlipidemia [56]. Other studies also informed that blood type A was linked with LDL cholesterol and higher total cholesterol, but there is no relation with HDL cholesterol [7, 57]. The most exciting discovery was the relationship of ABO and secretor blood types with serum levels of intestinal alkaline phosphatase (I-ALP) and apo lipoprotein B-48 (apo B-48); I-ALP is necessary for the passage of chylomicrons from bowels to the circulation and is thus an indicator for chylomicron absorption, whereas apo B-48 is a protein that strengthens the chylomicron membrane and is thus an indicator for chylomicron fabrication [7, 60]. There are important variations in serum I-ALP and apo B-48 between blood type O and B secretors and all other blood types; the O and B secretors have very high serum levels of these indicators relative to blood type A/AB secretors and nonsecretors of all blood types [7, 60]. ABO nonsecretors alone have about 20% of the serum I-ALP of secretors, and among secretors, blood type A has very low activity (2.8 ± 1.1 IU/L; mean ± SEM) compared to blood types B and O (14.1 ± 1.1 IU/L and 19.0 ± 2.5 IU/L, respectively) [7]. It is believed that I-ALP is linked with ABO antigens on RBCs of nonsecretors and is also accumulated by the A antigens of secretors, thus being quickly removed from circulation in these persons, while the soluble circulating antigens of O and B secretors favorably link with I-ALP and protect its removal in these persons [60]. Blood type A persons also have lesser serum apo B-48 levels, which can be due to a genetic downregulation of I-ALP activity in their intestines, subsequently lower chylomicron secretion [7, 60], and possibly lesser serum cholesterol levels.

#### 3.5.3. Diabetes Mellitus

A huge prospective study conducted in France indicates that there was no connection between the Rh blood type and risk of type 2 diabetes mellitus (T2DM). However, individuals with blood type O shows the lowermost risk of T2DM, whereas those with blood type B were at the uppermost risk, followed by type AB and type A individuals; nevertheless, the risk for type AB people did not have statistical implication [7, 61]. When Rh and ABO types were assessed together, blood type B^+^ persons showed the uppermost risk, followed by type AB^+^, A^–^, and A^+^ persons, but similar risk was seen for the other types [7, 61]. When adjusted for metabolic covariates (fasting blood glucose and lipids), blood type AB persons showed the maximum risk of T2DM (odds ratio, 1.95), followed by type B (odds ratio, 1.26) and type A (odds ratio, 1.21) as compared with blood type O persons, who had the lowest risk [61]. Other findings also indicated inconsistent results: a study in Yemen indicated that the maximum arbitrary blood sugar and insulin levels were found in blood type A, whereas blood type AB showed a defensive effect [56]. A research in Iraqi persons indicated greater blood glucose, total cholesterol, and blood pressure in blood type O persons, followed by lesser risk in type A, B, and AB persons, who showed the bottommost risk [7, 61]. A large study in Bangladesh indicated that there was no relationship between ABO blood types and T2DM. A study in Malaysia also shows lesser risk of T2DM in blood types A and O [62]. It has also been reported that nonsecretors are more likely to have T2DM [7, 57]. Undoubted confirmation has described a genetic relationship in those of European ancestry between nonsecretor status (se/se; homozygous for the A/A alleles of the *FUT2* gene) and insulin-dependent type 1 diabetes mellitus (T1DM) [7]. The odds ratio for nonsecretor status was 1.29 (*p* = 7.3 × 10^−14^) in the case of the control population, while the relative risk for nonsecretor status was 1.22 (*p* = 6.8 × 10^−6^) in the diabetic family population and the combined results clearly indicated a locus for T1DM in the *FUT2* gene (*p* = 4.3 × 10^−18^) [7]. The I-ALP findings by blood type for diabetes were the same as the findings for hyperlipidemia, with considerably greater serum I-ALP and entire ALP levels in blood type B and O secretors (together with controls) when compared to A secretors or ABO nonsecretors, but no substantial variation in serum I-ALP or entire ALP between A secretors and ABO nonsecretors (together with controls) [7, 33]. However, when comparing blood type B and O secretor diabetics to controls, I-ALP activity was similar between type 1 and type 2 diabetics but considerably greater in both types than in the controls; likewise, there was insignificant variation in I-ALP activity between type 1 and type 2 diabetics in the A secretor and ABO nonsecretor groups, but both types of diabetics had greater I-ALP activity than the controls in these groups [7, 33]. Further comparisons were made between the diabetics and controls in the group B and O secretors: mostly, fasting I-ALP activity was greater in the diabetics than in the controls; both types of diabetics had considerably greater liver ALP activity than the controls; type 2 diabetics had greater liver ALP than type 1 diabetics; and type 2 diabetics also had additional abnormal ALT and GGT values than type 1 diabetics [7, 33]. Troubled liver function could weaken the clearance of I-ALP by the liver and would elucidate the greater ALP levels found in the diabetics; high I-ALP has been described in victims with cirrhosis of the liver [7].

### 3.6. Malaria

The connection between the ABO blood type and malaria was first suggested by [1, 63]; they indicate that type B provides a selective advantage to malarial disease. By 1978, a noticeable number of type A patients were known from combined data analysis as compared to types B and O. However, malaria types were rarely stated in the literature [9]. According to the ABO phenotype, there are some variations in Lewis antigen level among A, B, and AB blood types, since these blood types carry fewer Le antigens than O types, because their corresponding transferases use similar precursors [1, 64]. The latest assumption has stated that *P*. *falciparum* malaria can be the reason for the three-fold greater incidence of the Le (a_b_) phenotype among people of African ancestry [25]. However, ABO blood types' role to defend against malaria has not been given much consideration; it has been revealed that blood type O can provide confrontation to severe *P. falciparum* malaria by reducing rosetting mechanisms [1, 65]. Severe malarial pathogenesis was linked to rosetting and sequestration. The first implies the formation of masses of diseased RBCs and normal RBCs and/or platelets. The second implies the course whereby *P. falciparum*-infected RBCs move on and stick to the microvascular endothelium and then are removed from the circulation [9]. The microcirculatory blockade by cytoadhesion can decrease oxygen and substrate supplies [9]. The complement receptor type 1 (CR1) is a rosetting receptor in RBCs, and CR1 seems to carry the Knops blood type antigens with the S1 (a) phenotype creating less rosettes [66]. Identification of a carbohydrate structure found in glycophorin A or B that consists of sialic acid and galactose is the major requisite for the entrance of *Plasmodium falciparum* merozoites into human RBCs [1, 67]. Sialic acid is common to some pathways that use glycophorins as ligands have also been related to ABO phenotypes. Sialic acid is one of the vital molecules for parasite attachment, and it has been thought that the human-chimpanzee variations in malaria vulnerability are linked to the genetic alterations in sialic acid molecules found in glycophorins [68]. In areas extremely endemic for *Plasmodium falciparum* malaria, it is well known that a variety of RBC polymorphisms related to confrontation to severe illness have experienced positive selection [69]. Overall, *P. vivax* accounts for 40% of cases [70]. The Duffy blood type system, which has 6 discrete antigens, is involved in *P. vivax* infections; mutations of the FY gene that result in RBCs without the Duffy antigen protect against infection by this strain of malaria [70].

## 4. Conclusions

Even though many studies have proven the association between ABO blood types and diseases by describing possible mechanisms, others did not confirm it and making the exact decision falls into uncertainty due to inconsistent results. Nevertheless, evidences were collected here to make this supposition clear. ABO may influence the risk of different diseases by different known and unknown mechanisms. It is now clear that ABO blood types are not the exact cause of diseases, but they can be susceptible and surrender to disease and health problems. In general, non-O blood types are more susceptible to diseases than O. It can be useful to increase the knowledge of persons in this aspect because individuals with high risk blood types could be screened and trained for modifying their lifestyles, health behavior, and environment and other attempts that may increase public health. The importance of human blood types can be seen more clearly in the context of population movement and the persistent combat between humans and infectious disease. Evidence for selection by infectious diseases at the level of the ABO and secretor genes is persuasive, but for other blood group antigens, founder effects appear more likely to account for the distribution of blood group polymorphisms except for parts of the world in which malaria is endemic. Available data suggests that survivals from malaria have been the most significant selective force acting on the blood groups. Moreover, further investigations have to be made particularly on the molecular level of ABO blood groups and their association with various diseases.
